# AMIC for traumatic focal osteochondral defect of the talar shoulder: a 5 years follow-up prospective cohort study

**DOI:** 10.1186/s12891-021-04506-z

**Published:** 2021-07-24

**Authors:** Christian Götze, Christian Nieder, Hanna Felder, Christian Dominik Peterlein, Filippo Migliorini

**Affiliations:** 1 Department of Orthopaedic Surgery, Auguste-Viktoria Clinic, Ruhr University Bochum, 32545 Bad Oeynhausen, Germany; 2 Department of Orthopaedics and Trauma Surgery, University Clinic Aachen, RWTH Aachen University Clinic, 52064 Aachen, Germany; 3 Department of Orthopaedicand Trauma Surgery, RWTH Aachen University Hospital, Pauwelsstraße 31, 52074 Aachen, Germany

**Keywords:** Talus, Osteochondral defects, AMIC

## Abstract

**Background:**

Autologous Matrix-Induced Chondrogenesis (AMIC) is addressed to osteochondral defects of the talus. However, evidence concerning the midterm efficacy and safety of AMIC are limited. This study assessed reliability and feasibility of AMIC at 60 months follow-up. We hypothesize that AMIC leads to good clinical outcome at midterm follow-up.

**Methods:**

Surgeries were approached with an arthrotomy via malleolar osteotomy. A resorbable porcine I/III collagen membrane (Chondro-Gide®, Geistlich Pharma AG, Wolhusen, Switzerland) was used. Patients were followed at 24 and 60 months. The primary outcome of interest was to analyse the Foot Function Index (FFI), and the subscale hindfoot of the American Orthopaedic Foot and Ankle Score (AOFAS). Complications such as failure, revision surgeries, graft delamination, and hypertrophy were also recorded. The secondary outcome of interest was to investigate the association between the clinical outcome and patient characteristics at admission.

**Results:**

Data from 19 patients were included. The mean age at admission was 47.3 ± 13.2 years, and the mean BMI 24.1 ± 4.9 kg/m^2^. 53% (10 of 19 patients) were female. At a mean of 66.2 ± 11.6 months, the FFI decreased at 24-months follow-up of 22.5% (*P* = 0.003) and of further 1.3% (*P* = 0.8) at 60-months follow-up. AOFAS increased at 24-months follow-up of 17.2% (*P* = 0.003) and of further 3.4 (*P* = 0.2) at 60-months follow-up. There were two symptomatic recurrences within the follow-up in two patients. There was evidence of a strong positive association between FFI and AOFAS at baseline and the same scores last follow-up (*P* = 0.001 and *P* = 0.0002, respectively).

**Conclusion:**

AMIC enhanced with cancellous bone graft demonstrated efficacy and feasibility for osteochondral defects of the talus at five years follow-up. The greatest improvement was evidenced within the first two years. These results suggest that clinical outcome is influenced by the preoperative status of the ankle. High quality studies involving a larger sample size are required to detect seldom complications and identify prognostic factors leading to better clinical outcome.

**Level of evidence:**

II, prospective cohort study.

## Introduction

Up to 50% of patients who experienced an acute ankle sprain demonstrated chondral damage [1]. If left untreated talar chondral defects can lead to early onset osteoarthritis [2]. Several surgical strategies have been addressed to chondral defects of the talus [3]. In 2005, Behrens et al. [4] described an enhanced microfractures technique wihch later has developed into the Autologous Matrix-Induced Chondrogenesis (AMIC). AMIC is a one-step surgical strategy which exploit the potential of autologous bone marrow-derived mesenchymal stem cells (BM-MSCs) [5, 6]. During AMIC the chondral defect is debrided until viable shoulders are achieved. Afterwards, bone marrow stimulation is performed to promote subchondral BM-MSCs migration in the chondral layer [7, 8]. A cell-free resorbable membrane is then trimmed and ensured over the defect, to create a blood clot rich in stem cells [9]. Several studies which focused on AMIC for chondral defects of the talus demonstrated high rate of patient satisfaction [10–24]. However, evidence concerning the midterm efficacy and safety of AMIC are limited. Recently, we published our results of AMIC on a cohort of 24 patients for chondral defects of the talus [5]. At 24 months follow-up all the patient reported outcome measures (PROMs) were significantly improved over their minimal clinically important difference (MCID). No patient experienced a complication. The present study assessed reliability and feasibility of AMIC on the same cohort of patients at 60 months follow-up. We hypothesize that AMIC leads to good clinical outcome at midterm follow-up.

## Material and methods

### Study Design

The present study was conducted according to the Consolidated Standards of Reporting Trials: the CONSORT statement [25]. The procedures reported in the present investigation were approved by the Ethics Committee of the Medical Faculty of the Ruhr University of Bochum, Germany (EK 2017–164). This study has been conducted according to the principles expressed in the Declaration of Helsinki. All patients were able to understand the nature of their treatment and provided written consent to use their clinical and imaging data for research purposes.

### Eligibility criteria

In the period 2013 until 2016 all patients who underwent AMIC as management for osteochondral defects of the talar shoulder were enrolled prospectively. Patients were recruited at the Auguste Viktoria Clinic in Bad Oeynhausen, Germany. The inclusion criteria were: (1) symptomatic chronic ankle pain, (2) previous failed conservative management, (3) evidence of osteochondral defect of the talar shoulder on MR, (4) focal defect, (5) traumatic onset, (6) lesion > 1.0 cm^2^ (7) patients aged 18 to 65 years, (8) minimum 60 months follow-up. The exclusion criteria were: (1) metabolic arthropathies, (2) kissing lesions, (3) large non-reconstructable defects, (4) uncorrectable axial deformity, (5) chronic inflammatory systemic disease, (6) body mass index (BMI) > 30 kg/m^2^, (7) bilateral ailments.

### Surgical technique

All the surgeries were performed in the same fashion by two surgeons (CG, CN) who were well beyond their learning curve. The surgical procedure was performed in isolation in a standardized fashion [5]. Briefly, all patients were approached with an arthrotomy via malleolar osteotomy. The osteochondral lesions were identified and debrided from the avital tissue until a viable shoulder was reached. Subchondral defect was filled with autologous cancellous bone graft harvested from the osteotomy site or from the ipsilateral iliac crest. An aluminium template was trimmed according to the lesion size. A type resorbable porcine I/III collagen membrane (Chondro-Gide®, Geistlich Pharma AG, Wolhusen, Switzerland) was trimmed according to the aluminum template and hydrated in a saline solution. Microfractures of 4 mm depth were performed into the defect using 1.2- or 1.4-mm K-wire under constant irrigation to avoid thermal necrosis. Afterwards, the membrane was placed into the defect and glued with fibrin (Tisseel, Baxter GmbH, Germany) to ensure fixation. Osteotomy was fixed with two malleolar screws. For the first six postoperative weeks patients were invited to wear a vacopedis boot, weight-bearing on the operated ankle was not allowed, and started passive range of motion exercises. After six weeks, patients started an intensive rehabilitation program, gradually increasing the weight load to full bearing, strengthening of the lower leg muscles, and proprioception training.

### Outcomes of interest

Patient baseline was recorded at admission: age, gender, side, size of defect, BMI, duration of symptoms. Patients were followed at 24 and 60 months. The primary outcome of interest was to analyse the Foot Function Index (FFI) [26], and the subscale hindfoot of the American Orthopaedic Foot and Ankle Score (AOFAS) [27]. Complications such as failure, revision surgeries, graft delamination, and hypertrophy were also recorded. Failure was defined as persistent pain which affects negatively the quality of life, limiting the participation to recreational activities. The secondary outcome of interest was to investigate the association between the clinical outcome and patient characteristics at admission.

### Statistical analysis

All statistical analyses were performed by one author (FM). The software STATA/MP 14.1 (StataCorp, College Station, TX) was used for the analyses. The Saphiro-Wilk test was performed to investigate data distribution. Mean and standard deviation (SD) were adopted for parametric data, with Student t test to assess significancy. For non-parametric data, median and interquartile range (IQR) were used, with Mann–Whitney U test for the significancy. A multivariate analysis was performed to assess associations between FFI and AOFAS at baseline and at last follow-up. A multiple linear model regression analysis using the Pearson Product-Moment Correlation Coefficient ($r$) was used. According to the Cauchy–Schwarz formula values of *r* tending to + 1 were considered as positive linear correlation and those to − 1 as negative. Values of 0.1 <|$r$ |< 0.3, 0.3 <|$r$ |< 0.5, and |$r$ |> 0.5 were considered to have respectively weak, moderate, and strong correlation. The overall significance was performed through the χ^2^ test. Values of *P* < 0.05 were considered statistically significant.

## Results

### Recruitment process

**Fig. 1:**
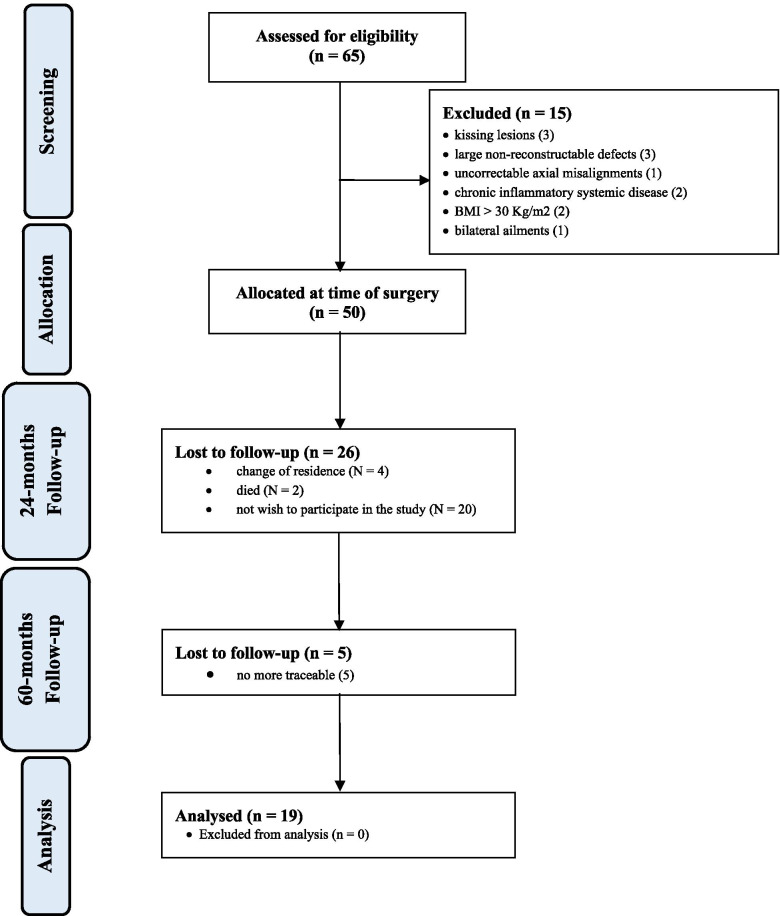
Flow-chart of the recruitment process

A total of 65 patients were enrolled. Further 15 patients were not eligible: kissing lesions (3), large non-reconstructable defects (3), uncorrectable axial deformity (1), chronic inflammatory systemic disease (2), BMI > 30 kg/m^2^ (2), bilateral ailments (1). 50 patients underwent AMIC in the period 2013–2016. At 24-months follow-up, 26 patients were not eligible: change of residence (*N* = 4), died (*N* = 2), not wish to participate in the study (*N* = 20). At 60 months follow-up, five patients were no more traceable. Finally, 19 patients were analysed at 60 months follow-up. The flow-chart of the recruitment process is shown in Fig. 1.

### Patient demographics

The mean length of the follow-up was 66.2 ± 11.6 months. The mean length of symptoms before the surgical intervention was 21.9 ± 30.3 months. The mean age at admission was 47.3 ± 13.2 years and the mean BMI 24.1 ± 4.9 kg/m^2^. 53% (10 of 19 patients) were female. The left side was involved in 37% (7 of 19) of patients. All lesions were located along the medial shoulder. The mean defect size was 6.9 ± 2.2 cm^2^. Study demographic of is shown in Table 1.

**Table 1:** Demographic of the patients at baseline

Endpoint	Value at baseline
Number of procedures	19
Mean age	47.3 ± 13.2
Female gender	53% (10 of 19)
Left side	37% (7 of 19)
Mean BMI	24.1 ± 4.9
Mean defect size (*cm*^*2*^)	6.9 ± 2.2
Mean duration of prior symptoms (*months*)	21.9 ± 30.3
Mean length of follow up (*months*)	66.2 ± 11.6

### Outcomes of interest

FFI decreased at 24-months follow-up of 22.5% (*P* = 0.003) and of further 1.3% (*P* = 0.8) at 60-months follow-up (Table 2).

**Table 2:** Results of FFI

Baseline – 24 months			24 – 60 months			Baseline – 60 months		
**MD**	**95% CI**	***P***	**MD**	**95% CI**	***P***	**MD**	**95% CI**	***P***
-22.5	8.064 to 36.936	0.003	-1.3	-12.110 to 14.710	0.8	23.8	11.343 to 36.257	0.0004

**Fig. 2:**
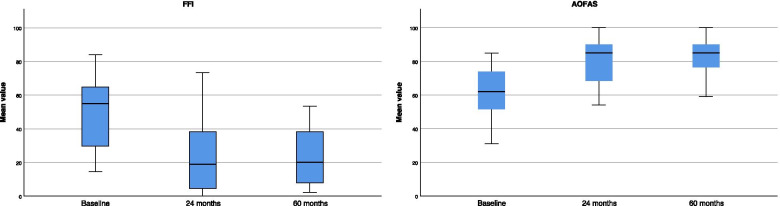
Box plots of FFI and AOFAS scores

AOFAS increased at 24-months follow-up of 17.2% (*P* = 0.003) and of further 3.4 (*P* = 0.2) at 60-months follow-up (Table 3). The Fig. 2 shows the box plots of the FFI and AOFAS scores.

**Table 3:** Results of AOFAS

Baseline – 24 months			24 – 60 months				Baseline – 60 months		
MD	95% CI	*P*	MD	95% CI	*P*	MD		95% CI	*P*
17.2	28.222 to 6.178	0.003	3.4	13.060 to -6.260	0.5	-17.2		30.223 to 10.977	0.0001

There were two failures in two females. Both patients evidenced on MRI a recurrence of chondral defect. One patient underwent a further AMIC at three years postoperatively due to growing ankle pain. At last follow-up she declared herself satisfied with the surgery. Another patient complained growing ankle pain and is currently waiting for a revision surgery.

The multivariate analysis evidenced a strong positive association between FFI at baseline vs 24 months follow-up (*r* = 0.9; *P* < 0.0001), baseline vs 60 months follow-up (*r* = 0.7; *P* = 0.001), 24 vs 60 months follow-up (*r* = 0.7; *P* = 0.002). The AOFAS at baseline demonstrated a strong positive association with the AOFAS at 24 months follow-up (*r* = 0.7; *P* = 0.0008), 60 months follow-up (*r* = 0.8; *P* = 0.0002), and between 24 vs 60 months follow-up (*r* = 0.7; *P* = 0.002). These results are shown in greater detail in Table 4.

**Table 4:** Multivariate analyses

Endpoint	Baseline—24 months		24—60 months		Baseline—60 months	
	***r***	***P***	***r***	***P***	***r***	***P***
**FFI**	0.9	< 0.0001	0.7	0.002	0.7	0.001
**AOFAS**	0.7	0.0008	0.8	0.0002	0.7	0.002

## Discussion

According to the main findings of the present study, AMIC enhanced with cancellous bone graft demonstrated efficacy and feasibility for osteochondral defects of the talus. The greatest efficacy was evidenced within the first two years, and no further difference was found from two to five years follow-up. Moreover, results of the present study suggest that clinical outcome is strongly influenced by the preoperative status of the ankle.

Several surgical strategies have been addressed to manage chondral defects of the talus [28, 29]. Isolated bone marrow stimulation (e.g. microfractures) is performed for smaller defects [9, 30, 31]. Microfractures are of simple execution, cost-effective, and achieved in a fully arthroscopic fashion. For bigger defects, osteochondral autograft transplantation has been widely performed [32, 33]. Osteochondral allograft transplantation has been introduced to avoid chondrocytes harvesting [34–36]. However, osteochondral allograft transplantation evidenced higher costs and rate of failure [37–40]. Autologous chondral transplantation (ACI) has been widely addressed to talar chondral defects [41, 42]. However, ACI requires a harvest site, two surgical sessions, and external chondrocytes expansion [43, 44]. Differently, AMIC is a single session strategy which spares chondrocytes harvesting and avoids cell expansion, exploiting the regenerative potential of BM-MSCs [5, 45]. These features commit AMIC of special interest of both patients and surgeons. Several studies showed promising results of AMIC for talar chondral defects, with high patient satisfaction and quick return to sport [10–24]. We identified only three long term studies investigating efficacy and feasibility of AMIC [12, 15, 21]. Becher et al. [12] in their clinical study followed 13 patients during five years follow-up. They reported a considerable reduction of the visual analogue scale (VAS) and improvement of the Hannover Scoring System. Furthermore, comparing AMIC with a matched group of patients who underwent isolated bone marrow stimulation, they evidenced no difference at five years follow-up [12]. Weigelt et al. [21] evaluated AMIC on a cohort of 33 patients at 4.7 years follow-up. At last follow-up the AOFAS scored 93/100 and the VAS (0–10) 1.4/10 [21]. They reported one case delayed union which did not require revision. 14 of 33 patients (42%) required screws removal. Further, 4 ankle arthroscopies (12% of patients) were performed because of limited motion or impingement, and one patient (3%) required supplementary gastrocnemius recession [21]. Gottschalk et al. [15] evaluated AMIC on 21 patients at one and five years follow-up. They evidenced a reduction of 23% of the FFI at one year postoperatively, along with a further nonsignificant decrease of 9% at five years follow-up [15]. Moreover, they performed a linear regression to investigate factors influencing the surgical success, concluding that the clinical outcome is strongly influenced by the preoperative status [15]. The latter was also evidenced by D’Ambrosi et al. [13] on 31 AMIC procedures. At two years follow-up they demonstrated an association between PROMs at baseline and the same at the follow-ups, concluding that the clinical results are strongly influenced by the preoperative status [13]. On the other hand, for patients with lesions of the central dome, the lesion size was proportionally associated with BMI, and that older patients had worse outcomes [46].

We used a malleolar osteotomy to distract the tibiotalar joint as standard. Malleolar osteotomy is associated to potential bony complications, loss of osteotomy reduction, delayed union or nonunion, symptomatic osteotomy site which may require removal [47, 48]. The osteotomy might impair the articular surface: given the limited regenerative potential of cartilage, it may lead to premature osteoarthrosis of the tibiotalar joint. Tibiotalar distraction with plantar flexion and Hintermann spreader is also widely used to access the joint cavity. Although it allows to faster weightbearing and recovery, it may predispose to soft tissue damage, especially the neurovascular structures [49–55]. Whether malleolar osteotomy perform better than plantar flexion and Hintermann spreader has not yet been clarified.

Currently, cell therapies assembled in synthetic polymers are of special interest for chondral regeneration. Chondral procedures have been enhanced with cell therapies to improve their regenerative potential. Bone marrow aspirate concentrate has shown promising results in clinical and preclinical studies [56–61]. Mesenchymal stem cells have also been employed to augment chondral procedures with promising outcomes [62–66]. The current literature evidences a high ratio of preclinical to clinical studies on this topic, suggesting that we are in a transition phase to human application. Many synthetic polymers are currently available or under experimentation as potential scaffolds, such as polyglycolic acid (PGA), polylactic acid (PLA), polylactic-co-glycolic acid (PLGA) and polyethylene glycol-terephthalate/ polybutylene terephthalate (PEOT/PBT) [67, 68].

This study has several limitations. The limited number of patients and the lack of randomisation may have biased the conclusion. Another important limitation was the limited recruitment rate. Indeed, from 50 patients who initially underwent AMIC, only 19 were retrieved at five years follow-up. The institution in which the operations were conducted, is a well-known centre for orthopaedic surgery, thus attracting patients from the whole country. Therefore, many patients did not agree to engage a long trip for research purposes. Unfortunately, the last follow-up was conducted during the COVID pandemic, which has considerably limited patient adhesion and participation to the survey. Two patients were secondary and tertiary referrals to our centre. These patients were not aware of their previous treatments and they have no documentation. All the patients underwent previous conservative management; however, given the heterogeneous nature and/or the lack of documentation on the previous conservative management, it was not possible to analyse them in a separate fashion. In this respect, results may not be fully reliable. The lack of a control group and the unblinded design of the study represent additional limitations. These limitations affect considerably the reliability of the conclusions; therefore, results of the present study must be interpreted with caution. Further studies should investigate the potential of AMIC in a larger scale, comparing them with other more recently regenerative strategies, such as hydrogels or synthetic polymers [67–70].

## Conclusion

AMIC enhanced with cancellous bone graft demonstrated efficacy and feasibility for osteochondral defects of the talus. The greatest improvement was evidenced within the first two years, and no further difference was found from two to five years follow-up. Moreover, results of the present study suggest that clinical outcome is strongly influenced by the preoperative status of the ankle.

## Data Availability

All data generated or analysed during this study are included in this published article.

## Abbreviations

AMIC: Autologous Matrix-Induced Chondrogenesis
FFI: Foot Function Index
AOFAS: American Orthopaedic Foot and Ankle Score
PROMs: Patient Report Outcome Measures
MRI: Magnetic Resonance Imaging
VAS: Visual Analogue Scale
